# The impact of preoperative oropharyngeal microflora, decontamination, and postoperative nosocomial and opportunistic infections on the occurrence of respiratory complications in patients undergoing esophagectomy for esophageal cancer after chemoradiotherapy. A single-center cohort

**DOI:** 10.1007/s00423-026-03966-y

**Published:** 2026-01-17

**Authors:** T. Řezáč, R. Vrba, M. Stašek, P. Špička, D. Klos, P. Zbořil

**Affiliations:** 1 Department of Surgery I, University Hospital Olomouc, Zdravotníku 248/7, Olomouc, 77900 Czech Republic; 2 Department of Surgery I, Faculty of Medicine and Dentistry, Palacky University Olomouc, Hněvotínská 976/3, Olomouc, 77515 Czech Republic

**Keywords:** Oesophagectomy, Mortality, Morbidity, Respiratory complications, Microbiome, Nosocomial infection, Opportunistic infection

## Abstract

**Introduction:**

Surgical oesophagectomy remains the primary curative treatment for oesophageal cancer. It is a challenging procedure that carries the possibility of serious postoperative complications.

**Methodology:**

Retrospective analysis of preoperative oropharyngeal microbiologic cultivation, the role of decontamination, and the postoperative microbiologic cultivation in patients after hybrid esophagectomy with mild or severe respiratory complications (pneumonia and respiratory failure), and anastomotic leakage.

**Results:**

Significantly more pneumonias were found in the group without eradication (Escherichia coli, facultative anaerobe, p=0.016) and in the group with new infection (Pseudomonas aeruginosa (hospital-acquired infection, p<0. 0001), Enterobacter cloacae (facultatively anaerobic, p=0.032), E. coli (facultative anaerobes, p<0.0001), Klebsiela pneumoniae (hospital-acquired infection, p<0.0001), Staphylococcus aureus (facultative anaerobe, p=0.0001), Acinetobacter junii (nosocomial infection, p=0.032), and Candida albicans (opportunistic pathogenic yeast, p<0.0001)) p<0.0001. Postoperative positivity for Citrobacter freundi increased the odds of leak by 10.76 times (facultative anaerobic, p=0.042, OR 10.76), and for E. coli by 3.17 times (p=0.017, OR 3.17).

**Conclusion:**

Despite the use of targeted antibiotic therapy, a significant proportion of opportunistic and nosocomial infections still occur in the pattern of postoperative respiratory and anastomotic complications in patients after esophagectomy.

**Supplementary Information:**

The online version contains supplementary material available at 10.1007/s00423-026-03966-y.

## Abstract (unstructured)

The article evaluates the importance of preoperative targeted microbiological decontamination in esophagectomy and the prevention of nosocomial infection. Nosocomial and opportunistic infections are significant sources of pulmonary complications and septic conditions, which can be prevented to improve postoperative morbidity.

**Supplementary Information:**

The online version contains supplementary material available at 10.1007/s00423-026-03966-y.

## Introduction

Surgical oesophagectomy remains the primary curative treatment for oesophageal cancer, despite the availability of multidisciplinary treatment. Significant therapeutic progress has been made in recent decades. The diagnostic algorithm for the disease has been improved by using new diagnostic modalities such as endosonography (EUS) and positron emission computed tomography (PET/CT). Additionally, minimally invasive (MIE) or robotic-assisted minimally invasive esophagectomy (RAMIE) can be used. Pre- and postoperative nutritional care is important, as is the implementation of an Enhanced Recovery After Surgery (ERAS) protocol. The mortality rate following oesophagectomy has significantly decreased, particularly in centres with a high volume of patients. Fuchs found that patients treated in high-volume centres (over 20 esophagectomies per year) had a significantly lower mortality rate of 4.01% compared to 8.39% in medium-volume centres (6 to 19 esophagectomies) and 11.4% in centres performing under six esophagectomies per year [1].

Despite treatment advances, the procedure still carries a relatively high morbidity rate compared to other gastrointestinal tract malignancies. Esophagectomy mortality is mainly caused by major pulmonary complications, such as respiratory failure and acute respiratory distress syndrome (ARDS) [2].

This report focuses on patients who experienced postoperative respiratory complications, such as pneumonia or respiratory failure, as well as those with an anastomotic leak. The analysis evaluated the impact of the oropharyngeal microbiome before surgery on postoperative complications, as well as the effect of selective decontamination on postoperative culture. It also assessed the role of individual bacterial or fungal species in complications related to nosocomial and opportunistic infections.

## Method

### Study design

A retrospective analysis of our computerised institutional registry was performed. The study included patients who underwent curative esophagectomy for tumour at the University Hospital Olomouc, a complex oncosurgical centre, between January 2015 and December 2022. Patients who underwent palliative stenting, nutritional jejunostomy only, emergency surgery, surgery for benign tumour, generalisation or progression of the disease after oncological treatment, and patients with ASA IV classification were excluded from the study. The medical records of 215 patients who met the inclusion criteria were reviewed.

### Setting

This was a retrospective study on prospectively registered consecutive patients undergoing elective esophagectomy.

### Participants

The study was conducted following the Declaration of Helsinki, and all patients provided informed consent for the use of their data. All patients met the inclusion criteria and underwent clinical staging of the disease, which included endoscopic examination with tumor biopsy, histological examination, endosonographic examination, and PET/CT examination. Preoperative examinations, such as spirometry, nutritional assessment, and anesthesiology evaluation (ASA I-III), were performed. An oropharyngeal secretion culture was taken one week before admission to provide targeted antibiotic prophylaxis or treatment. A control culture was taken on the first postoperative day (POD1). The surgeries were performed by upper gastrointestinal tract surgeons with over ten years of experience.

### Variables

The following clinical data were collected:

#### Patient background

Age, sex, personal medical history, ischemic heart disease, hypertension, diabetes mellitus, renal failure, chronic obstructive bronchial disease, ASA score, tumor localization, nutritional parameters (body mass index, total protein, albumin, prealbumine), alcohol consumption, smoking status and history, type of surgery.

#### Data related to surgery

Perioperative complications, blood loss, postoperative respiratory and septic complications, presence of leak, hospital stay and mortality.

#### Data related to cultivation

The results of preoperative and postoperative oropharyngeal cultivation, the success of ATB decontamination, pulmonary or septic complications.

### Bias

The source of potential bias is the relatively small sample size of patients with specific diagnoses and the potential for unintended patient selection due to the geographical location and the fact that the infection occurred in a single medical center.

### Statistical analysis

IBM SPSS Statistics version 23 statistical software (Armonk, NY: IBM Corp.) was used for data analysis. Quantitative parameters were compared using the Mann-Whitney U test, and the chi-square test or Fisher’s exact test was used to compare qualitative parameters. Survival analysis was performed through Kaplan–Meier statistics. Logistic regressions correlated each variable with the outcomes defined: pneumonia, respiratory failure. Variables considered clinically relevant were selected for multivariable logistic regression. The normality of the data was assessed using the Shapiro-Wilk test. All tests were done at a 0.05 level of significance.

## Results

### Participants

**Graph 1:**
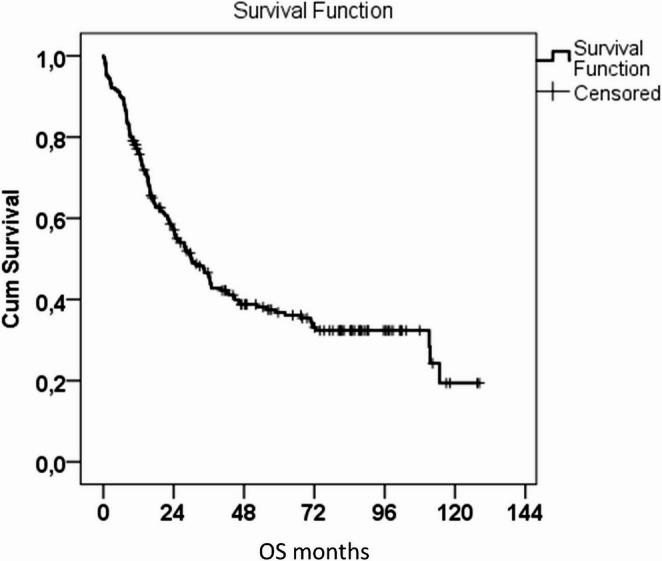
Kaplan-Meier analysis of patient survival in months

Two hundred and fifteen patients, 177 men and 38 women, met the inclusion criteria. Of these, 17% were regular alcohol consumers and over 40% were smokers. The most common comorbidities were hypertension (29.8%), coronary heart disease (18.6%), diabetes (11.6%) and chronic obstructive pulmonary disease (7.4%). Patients had a mean age of 62.3 ± 9 years and a mean BMI of 25.5 kg/m2 ± 4.6. All preoperative nutritional values were between normal, with total protein at 65.3 g/L ± 6.1, albumin at 39.4 ± 4.7 and pre-albumin at 0.189 ± 0.045. The mean survival of the patients was 54.5 months (95%CI: 47.4–6.6 months), with a median survival of 30.1 months (95%CI: 22.2–37.9 months). Tables 1 and 2, graph 1.

**Table 1:** General characteristics of the patient cohort

	Total *n* = 215	%
Sex		
Male	177	82.3%
Female	38	17.7%
Alcohol consumption		
No or occasionally	177	82.3%
Regularly	38	17.7%
Diabetes melitus		
No	190	88.4%
Yes	25	11.6%
Chronic obstructive pulmonary disease		
No	199	92.6%
Yes	16	7.4%
Coronary artery disease		
No	175	81.4%
Yes	40	18.6%
Renal failure		
No	207	96.7%
Yes	7	3.3%
Hypertension		
No	151	70.2%
Yes	64	29.8%
Smoking status		
No	126	58.6%
Yes	89	41.4%
ASA score		
1	9	4.2%
2	132	61.4%
3	74	34.4%
Age (years, mean, ±SD)	62.3	9.0
BMI (kg/m2, mean, ±SD)	25.5	4.6
Total protein (g/L, mean, ±SD)	65.3	6.1
Albumin (g/L, mean, ±SD)	39.4	4.7
Prealbumin (g/L, mean, ±SD)	0.189	0.045

**Table 2:** Kaplan-Meier analysis of patient survival in months

Mean	95% Confidence Interval		Median	95% Confidence Interval	
	Lower Bound	Upper Bound		Lower Bound	Upper Bound
54.5	47.4	61.6	30.1	22.2	37.9

### Main results

Preoperative oropharyngeal culture yielded 12 strains (graph 2), while sputum culture captured 19 strains (graph 3).

The impact of postoperative sputum culture results on the development of respiratory complications was found to be significant. Specifically, hospital-acquired infections of Pseudomonas aeruginosa (*p* < 0.0001) and Klebsiella pneumoniae (*p* < 0.0001), as well as facultatively anaerobic Enterobacter cloacae (*p* = 0.032) and E. coli (*p* < 0.0001), were identified as contributing factors. Staphylococcus aureus, a facultative anaerobe (*p* = 0.0001); Acinetobacter junii, a nosocomial infection (*p* = 0.032); and Candida albicans, an opportunistic pathogenic yeast (*p* < 0.0001), were identified (0001) (Table 3).

**Table 3:** A table for individual bacterial families

Postoperative cultivation of sputum		Cultivation				p-value
		Preoperative		Postoperative		
		No.:	%	No.:	%	
Stenotrophomonas maltophilia	0	99	100.0%	113	97.4%	0.251
	1	0	0.0%	3	2.6%	
Pseudomonas putida	0	99	100.0%	115	99.1%	1
	1	0	0.0%	1	0.9%	
Pseudomonas aeruginosa	0	98	99.0%	94	81.0%	**< 0.0001**
	1	1	1.0%	22	19.0%	
Burkholderia multivorans	0	99	100.0%	111	95.7%	0.063
	1	0	0.0%	5	4.3%	
Proteus mirabilis	0	99	100.0%	113	97.4%	0.251
	1	0	0.0%	3	2.6%	
Enterobacter asburiae	0	99	100.0%	114	98.3%	0.501
	1	0	0.0%	2	1.7%	
Enterobacter species	0	99	100.0%	107	92.2%	**0.004**
	1	0	0.0%	9	7.8%	
Enterobacter aerogenes	0	99	100.0%	114	98.3%	0.501
	1	0	0.0%	2	1.7%	
Enterobacter cloacae	0	99	100.0%	110	94.8%	**0.032**
	1	0	0.0%	6	5.2%	
E. colli	0	99	100.0%	96	82.8%	**< 0.0001**
	1	0	0.0%	20	17.2%	
Raoultella ornithinolytica/enterobacter	0	99	100.0%	114	98.3%	0.501
	1	0	0.0%	2	1.7%	
Citrobacter freundii	0	99	100.0%	112	96.6%	0.126
	1	0	0.0%	4	3.4%	
Klebsiella oxytoca	0	98	99.0%	112	96.6%	0.377
	1	1	1.0%	4	3.4%	
Klebsiela pneumonie	0	98	99.0%	91	78.4%	**< 0.0001**
	1	1	1.0%	25	21.6%	
Aggregatibacter segnis	0	99	100.0%	115	99.1%	1
	1	0	0.0%	1	0.9%	
Haemophilus parainfluenzae	0	98	99.0%	111	95.7%	0.221
	1	1	1.0%	5	4.3%	
Aspergillus fumigatus	0	99	100.0%	113	97.4%	0.251
	1	0	0.0%	3	2.6%	
Streptococcus anginosus	0	99	100.0%	113	97.4%	0.251
	1	0	0.0%	3	2.6%	
streptococcus species	0	98	99.0%	108	93.1%	**0.040**
	1	1	1.0%	8	6.9%	
Staphylococcus haemolythicus	0	99	100.0%	114	98.3%	0.501
	1	0	0.0%	2	1.7%	
Staphylococcus aureus	0	98	99.0%	97	83.6%	**0.0001**
	1	1	1.0%	19	16.4%	
Acinetobacter junii	0	99	100.0%	110	94.8%	**0.032**
	1	0	0.0%	6	5.2%	
Moraxella/Branham./catarrhalis	0	99	100.0%	115	99.1%	1
	1	0	0.0%	1	0.9%	
Aeromonas jandaei	0	99	100.0%	115	99.1%	1
	1	0	0.0%	1	0.9%	
Enterococcus sp.	0	99	100.0%	114	98.3%	0.501
	1	0	0.0%	2	1.7%	
Candida albicans	0	96	97.0%	77	66.4%	**< 0.0001**
	1	3	3.0%	39	33.6%	
Morganella morganii	0	99	100.0%	111	95.7%	0.063
	1	0	0.0%	5	4.3%	
Serratia marcescens	0	99	100.0%	104	89.7%	**0.001**
	1	0	0.0%	12	10.3%	

The study conducted Cox regression analysis and estimated adjusted survival values through multivariate analysis. The results confirmed a statistically significant impairment of survival in the presence of Morganella morganii (RR 4.408, *p* = 0.001) and Burkholderia multivorans (RR 3.999, *p* = 0.003) (Table 4).

**Table 4:** Estimation of adjusted values of the RR statistic using cox regression analysis. Results of multivariate analysis

Postoperative cultivation	*p*-value	RR	95,0% CI for RR	
			Lower	Upper
Staphylococcus aureus	**0.019**	**1.880**	1.108	3.191
Morganella morganii	**0.001**	**4.408**	1.779	10.923
Burkholderia multivorans	**0.003**	**3.999**	1.622	9.860

Univariate analysis revealed that Citrobacter freundii is an additional risk factor (RR 3.592, *p* = 0.013). Notably, the presence of a new infection alone increased the risk of mortality (RR 1.578, *p* = 0.009) (Table 5).

**Table 5:** Cox regression analysis was used to estimate unadjusted values of the risk ratio (RR) statistic. The results of the univariate analyses

Postoperative cultivation	*p*-value	RR	95,0% CI for RR	
			Lower	Upper
New infection	**0.009**	**1.578**	1.119	2.226
Staphylococcaceae family	**0.049**	**1.674**	1.003	2.795
klebsiela pneumonie	**0.035**	**1.654**	1.037	2.639
Staphylococcus aureus	**0.039**	**1.741**	1.030	2.945
Morganella morganii	**0.003**	**3.941**	1.598	9.723
Burkholderia multivorans	**0.005**	**3.586**	1.460	8.805
Citrobacter freundii	**0.013**	**3.592**	1.308	9.869

When evaluating the relationship between bacterial infection and anastomotic leak, the Cox regression analysis showed that postoperative positivity for E. coli increased the chance of leak by 3.17 times, Citrobacter freundi increased the chance of leak by 10.76 times, and the Enterobacteriaceae family increased the chance of leak by 3.02 times (Table 6).

**Table 6:** The table presents only the statistically significant results of the univariate analyses

Postoperative cultivation	*p*-value	OR	95% C.I.for OR	
			Lower	Upper
E colli	**0.017**	**3.170**	1.230	8.174
Citrobacter freundii	**0.042**	**10.76**	1.093	105.9
Enterobacteriaceae family	**0.001**	**3.024**	1.557	5.871

## Discussion

In general, the incidence of respiratory complications is considered an adverse prognostic factor [3, 4]. Pneumonia is a significant cause of post-esophagectomy mortality and worsens the long-term survival of patients. Therefore, controlling pneumonia is a crucial issue in postoperative esophagectomy management.

Preoperative treatments have been found to have a positive effect on postoperative mortality and morbidity and do not contribute to pulmonary complications, according to recent reports [5, 6].

The significant risk factors for postoperative complications following preoperative treatments include bone marrow suppression, mucositis, odynophagia, and anorexia. These factors may disrupt the immune and nutrition systems, leading to infectious complications [7].

Additionally, preoperative treatments may alter the bacterial flora of the sputum, increasing its complexity in cancer patients [8]. The normal flora provides the host with ecological barriers that prevent pathogen attacks. Therefore, a decrease in normal bacteria increases the risk of disease.

The relationship between bacterial culture and post-esophagectomy pneumonia is of great interest to upper gastrointestinal surgeons. However, only a few studies have investigated the bacterial culture of esophagectomy patients [9, 12].

In our study, patients with positive postoperative sputum and throat cultures had a significantly higher postoperative pneumonia rate. It has been widely accepted that oral bacteria are a significant source of bacteria that cause bacterial pneumonia. The aspiration of oral bacteria can cause postoperative pneumonia. Research has shown that dental plaque, the largest bacterial reservoir in the mouth, is a significant cause of aspiration pneumonia [10]. Recent studies have focused on the role of plaque in this condition.

El-Solh [10] demonstrated that preoperative dental plaque pathogens are risk factors for post-thoracotomy pneumonia in patients with thoracic esophageal cancer. He noted that the pathogens obtained by bronchoalveolar lavage were homogenous with those obtained from the dental plaque culture. Additionally, Akutsu [11] reported that dental brushing before surgery for esophageal cancer can reduce the incidence of postoperative pneumonia.

Kosumi [12] demonstrated a weaker association with pneumonia incidence compared to the dental plaque culture test. This finding suggests that the presence of pathogens cannot be comprehensively detected by plaque culture alone. Some patients may still have pathogens in their throat or sputum.

Matsui [13] reported that out of 238 patients who underwent esophagectomy for esophageal cancer, forty-one patients developed pneumonia within one week after surgery. The bacterial species significantly associated with pneumonia were Gram-positive cocci and Gram-negative rods.

Our study, moreover, found that 33.6% of patients had a positive culture for the yeast strain. Candida is usually a commensal organism, but it can cause disease in immunocompromised people, intensive care units, or patients with dysfunctional epithelial barriers. In Western countries, most people carry Candida species as commensals in their intestinal microbiota. Patients often exhibit an increase in Candida species during prolonged stays in the intensive care unit or following transplantation. Intra-abdominal candidiasis is more likely to develop in patients undergoing abdominal surgery or gastrointestinal tract pathology [14]. A challenging esophagectomy meets all of these criteria. The detection rate of Candidal infection is not negligible and has been reported in the literature to range from 19.4% to 63.8% [15, 16].

In a study of 609 patients who underwent esophagectomy for esophageal cancer, Yoshida [17] reported that the presence of sputum was associated with an increased risk of developing pneumonia, regardless of microbiological culture. On preoperative CT, sputum was present in the respiratory tract of 76 (12.5%) patients. This finding was significantly associated with older age, a more extreme smoking habit, worse performance status, lower forced expiratory volume, and more frequent pulmonary comorbidities. The incidence of postoperative pneumonia was higher in these patients (16%) than in those without sputum (8%) (*p* = 0.028). The presence of sputum in the main bronchus was associated with higher frequencies of morbidity according to the Clavien-Dindo (CD) classification (CD II *p* = 0.019, CD IIIb *p* = 0.058), pneumonia (*p* = 0.10), and pulmonary morbidity (*p* = 0.19) compared to sputum in the trachea alone. Multivariate analysis revealed that sputum in the respiratory tract was an independent risk factor (hazard ratio, 2.07; 95% confidence interval, 1.019–4.207; *p* = 0.044) for postoperative pneumonia.

It should be noted that Systemic Inflammatory Response Syndrome (SIRS) significantly contributes to the development of pneumonia, especially in patients with positive postoperative cultures. Multivariate analyses for clinical factors of postoperative pneumonia showed that SIRS was an independent predictor for pneumonia [15]. The production of numerous cytokines due to SIRS leads to increased adhesion factors, damage to lung tissue, and impaired host immunity, resulting in bacterial growth and pneumonia development. Additionally, there is a role in priming the inflammatory response. Priming is a transitional state in which neutrophils and macrophages become more responsive to activating stimuli. It is known that exposure to one stimulus enhances the cell’s ability to mount an enhanced activation response to a second individual stimulus [18]. Therefore, a mild infection can initiate a priming condition, and subsequent surgical stress can cause an exaggerated immune response, resulting in a more severe systemic inflammatory response syndrome (SIRS) in patients.

In our study, we evaluated 48 (41.4%) mild infections and 26 (22.4%) severe infections as SIRS from 116 patients with postoperative confirmed positive sputum cultivation. These results support the previous claim.

Currently, it is possible to influence preoperative culture without complications through targeted antibiotic treatment. By implementing the ERAS protocol, we can enhance the patient’s nutritional status and physical activity, preparing them for a demanding procedure. However, despite these efforts, there are factors that we cannot control. The patient is typically elderly, an active or former smoker and alcohol user, and burdened with neoadjuvant radiochemotherapy. This treatment significantly impairs their reserves and causes side effects such as bone marrow suppression, mucositis, odynophagia, and anorexia. All of these factors can negatively impact the patient’s immune and nutritional systems, potentially worsening their prognosis and increasing the risk of nosocomial infections. Infections that develop 48 h after hospital admission, regardless of whether they were present at the time of admission. While the patient’s condition certainly plays a role in their development, other factors also contribute. Linden’s meta-analysis [19] demonstrates that repeated reintubation is a significant negative prognostic factor, a predictor of patient mortality, and a potential cause of nosocomial infection. Isigi’s systematic review [20] confirmed as a risk factors older age, intrahospital transfers, cross-infection, longer hospital stays, readmissions, prior colonization with opportunistic organisms, comorbidities, and prior intake of antibiotics and urinary catheters. The role of blood groups in infection, as published by Zhong [21], is not well-known. Zhong’s study, which involved 300,000 participants, found that patients with blood groups B and AB were more susceptible to Escherichia coli (OR = 1.783, *p* = 0.039), while those with blood group A were more susceptible to Staphylococcus aureus (OR = 2.539, *p* = 0.019) and Pseudomonas aeruginosa (OR = 5.724, *p* = 0.003). Additionally, patients with blood groups A and AB were more susceptible to Pseudomonas aeruginosa (OR = 4.061, *p* = 0.008). The study found that the A group had a higher vulnerability to urinary tract infections (OR = 13.672, *p* = 0.019), while the B group had a higher vulnerability to skin and soft tissue infections (OR = 2.418, *p* = 0.016). The B and AB groups had a higher vulnerability to deep incision infections (OR = 4.243, *p* = 0.043). It is expected that preventing patient preparation, appropriate ATB prophylaxis, treatment of minor respiratory complications, and the development of major complications requiring reintubation will break the self-perpetuating cycle. This, in turn, may reduce the mortality rate of esophagectomy for pulmonary complications.

The aim for the future is to develop a universal and comprehensive algorithm for diagnosing and preparing patients, including preventing and treating all complications.

The report acknowledges the limitations of the study, which was retrospective and evaluated only at one center. A prospective study is needed to investigate alterations in the bacterial flora of sputum. However, the review included over 200 esophagectomies spanning eight years. It is also important to note that sputum monitoring was conducted in this study based on the physician’s preference, which may have biased the length of hospital stay. It has not been confirmed that monitoring sputum significantly improves the postoperative course due to the small number of patients with postoperative pneumonia. Further analysis is required to verify whether monitoring sputum culture improves the post-esophagectomy outcome.

The study has a final limitation related to the performance of esophagectomies based on tumor location using different modifications, such as the transthoracic and transhiatal approaches. It should be noted that the surgeries were performed by only three highly experienced esophageal surgeons with many years of experience in esophageal surgery, and the institution meets the criteria of a high-volume center. Therefore, the generalizability of the findings would be increased with data from more patients.

## Conclusion

Respiratory failure and acute respiratory distress syndrome (ARDS) can result in severe pulmonary complications that affect patient mortality and morbidity after esophagectomy. Managing minor pulmonary complications therapeutically can improve immediate postoperative outcomes and reduce the need for reintubation due to significant complications. However, there is room for improvement in postoperative outcomes and survival, especially during the preoperative period. This process includes preparing the patient for surgery, optimizing and stabilizing any underlying medical conditions, improving physical performance, enhancing nutrition, and implementing targeted antibiotic prophylaxis.

## Supplementary Information

Below is the link to the electronic supplementary material.

ESM 1 (DOCX 23.5 KB)

ESM 2 (DOCX 20.8 KB)

## Data Availability

The data that support the findings of this study are available in Open Science Framework, accesed on 17. jan 2024, https://osf.io/fdwa5/?view_only=5ac523364a144484997d478be8b85194.
