# Supplementary material for: The impact of preoperative oropharyngeal microflora, decontamination, and postoperative nosocomial and opportunistic infections on the occurrence of respiratory complications in patients undergoing esophagectomy for esophageal cancer after chemoradiotherapy. A single-center cohort
Source: Langenbecks Arch Surg. 2026 Jan 17;411(1):70. doi: 10.1007/s00423-026-03966-y (PMC12847148; doi:10.1007/s00423-026-03966-y)
Supplement: Supplementary file 1 — (DOCX 23.5 KB) [file 423_2026_3966_MOESM1_ESM.docx]

- **Acinetobacter junii** - This bacterium has been linked to nosocomial infections, including catheter-related bloodstream infections and cellulitis.
- [**Burkholderia multivorans**](https://www.sciencedirect.com/topics/medicine-and-dentistry/burkholderia-multivorans) - formally known as [Pseudomonas cepacia](https://www.sciencedirect.com/topics/medicine-and-dentistry/burkholderia-cepacia) is an aerobic, gram-negative bacillus, is a group of opportunistic [pathogens](https://www.sciencedirect.com/topics/medicine-and-dentistry/pathogen) that can be found in soil and water. There are 17 different and highly virulent species in the complex that are primarily associated with causing infections [in patients](https://www.sciencedirect.com/topics/medicine-and-dentistry/inpatient) with cystic fibrosis, [chronic granulomatous disease](https://www.sciencedirect.com/topics/medicine-and-dentistry/chronic-granulomatous-disease), and [immunosuppression](https://www.sciencedirect.com/topics/medicine-and-dentistry/immunosuppressive-treatment). Nosocomial infections have also been reported through contaminated anesthetic solutions, water sources, [medical devices](https://www.sciencedirect.com/topics/medicine-and-dentistry/medical-device), [disinfectants](https://www.sciencedirect.com/topics/medicine-and-dentistry/disinfectant-agent), and non-sterile medical products.
- **Candida albicans -** opportunistic [pathogenic yeast,](https://en.wikipedia.org/wiki/Pathogenic_yeast) that is a common member of the human [gut flora](https://en.wikipedia.org/wiki/Gut_flora). It can also survive outside the human body. Is detected in the gastrointestinal tract and mouth in 40–60% of healthy adults. It is usually a [commensal](https://en.wikipedia.org/wiki/Commensal) organism, but it can become [pathogenic](https://en.wikipedia.org/wiki/Pathogen) in [immunocompromised](https://en.wikipedia.org/wiki/Immunodeficiency) individuals under a variety of conditions. It is one of the few species of the genus [Candida](https://en.wikipedia.org/wiki/Candida_(fungus)) that cause the human infection [candidiasis](https://en.wikipedia.org/wiki/Candidiasis), which results from an overgrowth of the fungus. Candidiasis is, for example, often observed in [HIV](https://en.wikipedia.org/wiki/HIV)-infected patients. C. albicans is the most common fungal species isolated from [biofilms](https://en.wikipedia.org/wiki/Biofilm) either formed on (permanent) implanted medical devices or on human [tissue](https://en.wikipedia.org/wiki/Tissue_(biology)).
- **Citrobacter freundi -** facultative anaerobic [Gram-negative bacteria](https://en.wikipedia.org/wiki/Gram-negative_bacteria) of the family Enterobacteriaceae which currently consists of 13 recognized species.C. freundii is a soil-dwelling microorganism, but can also be found in water, sewage, food, and the intestinal tracts of animals and humans.
- **Enterobacter species -** a g[ram-negative](https://en.wikipedia.org/wiki/Gram-negative), [facultatively anaerobic](https://en.wikipedia.org/wiki/Facultative_anaerobic_organism), [rod-shaped](https://en.wikipedia.org/wiki/Bacillus_(shape)), non-spore-forming [bacteria](https://en.wikipedia.org/wiki/Bacteria) of the family [Enterobacteriaceae](https://en.wikipedia.org/wiki/Enterobacteriaceae). Several strains of these bacteria are [pathogenic](https://en.wikipedia.org/wiki/Pathogen) and cause [opportunistic infections](https://en.wikipedia.org/wiki/Opportunistic_infection) in [immunocompromised](https://en.wikipedia.org/wiki/Immunocompromised) (usually hospitalized) hosts and in those who are on [mechanical ventilation](https://en.wikipedia.org/wiki/Mechanical_ventilation). The [urinary](https://en.wikipedia.org/wiki/Urinary_tract) and [respiratory tracts](https://en.wikipedia.org/wiki/Respiratory_tract) are the most common sites of [infection](https://en.wikipedia.org/wiki/Infection).
- **Escherichia colli** - [facultative anaerobe](https://en.wikipedia.org/wiki/Facultative_anaerobe) constitute about 0.1% of [gut microbiota](https://en.wikipedia.org/wiki/Gut_microbiota), and [fecal–oral transmission](https://en.wikipedia.org/wiki/Fecal%E2%80%93oral_route) is the major route through which pathogenic strains of the bacterium cause disease.
- **Klebsiela pneumonie -** the most common condition caused is [pneumonia](https://en.wikipedia.org/wiki/Pneumonia), typically in the form of [bronchopneumonia](https://en.wikipedia.org/wiki/Bronchopneumonia) and also [bronchitis](https://en.wikipedia.org/wiki/Bronchitis). These patients have an increased tendency to develop lung [abscesses](https://en.wikipedia.org/wiki/Abscess), cavitation, [empyema](https://en.wikipedia.org/wiki/Empyema), and [pleural adhesions](https://en.wikipedia.org/wiki/Adhesion_(medicine)). It has a death rate around 50%, even with [antimicrobial](https://en.wikipedia.org/wiki/Antimicrobial) therapy.
- **Moraxellaceae** - [opportunistic infection,](https://en.wikipedia.org/wiki/Opportunistic_infection) usually resides in respiratory tract, but can gain access to the [lower respiratory tract](https://en.wikipedia.org/wiki/Lower_respiratory_tract) in patients with chronic chest disease or compromised host defenses, thus causing tracheobronchitis and [pneumonia](https://en.wikipedia.org/wiki/Pneumonia). For example, it causes a significant proportion of lower respiratory tract infections in elderly patients with [COPD](https://en.wikipedia.org/wiki/COPD) and [chronic bronchitis](https://en.wikipedia.org/wiki/Bronchitis#Chronic_bronchitis)
- **Morganella morganii** - a species of [Gram-negative](https://en.wikipedia.org/wiki/Gram-negative) [bacteria](https://en.wikipedia.org/wiki/Bacterium). It has a [commensal](https://en.wikipedia.org/wiki/Commensalism) relationship within the intestinal tracts of humans, mammals, and reptiles as normal flora. Although M. morganii has a wide distribution, it is considered an uncommon cause of community-acquired infection, and it is most often encountered in [postoperative](https://en.wikipedia.org/wiki/Postoperative) and other [nosocomial infections](https://en.wikipedia.org/wiki/Nosocomial_infection), such as [urinary tract infections](https://en.wikipedia.org/wiki/Urinary_tract_infections).
- **Pseudomonas aeruginosa** - a [multidrug resistant](https://en.wikipedia.org/wiki/Multiple_drug_resistance) pathogen recognized for its ubiquity, its [intrinsically](https://en.wikipedia.org/wiki/Intrinsic_and_extrinsic_properties) advanced [antibiotic resistance](https://en.wikipedia.org/wiki/Antibiotic_resistance) mechanisms, and its association with serious illnesses – [hospital-acquired infections](https://en.wikipedia.org/wiki/Hospital-acquired_infections) such as [ventilator-associated pneumonia](https://en.wikipedia.org/wiki/Ventilator-associated_pneumonia) and various [sepsis](https://en.wikipedia.org/wiki/Sepsis) [syndromes](https://en.wikipedia.org/wiki/Syndromes).
- **Staphylococcus aureus -** usual member of the [microbiota](https://en.wikipedia.org/wiki/Microbiota) of the body, frequently found in the [upper respiratory tract](https://en.wikipedia.org/wiki/Respiratory_tract) and on the [skin](https://en.wikipedia.org/wiki/Human_skin), and is a [facultative anaerobe,](https://en.wikipedia.org/wiki/Facultative_anaerobic_organism) andopportunistic pathogen, being a common cause of [skin infections](https://en.wikipedia.org/wiki/Skin_infection) including [abscesses](https://en.wikipedia.org/wiki/Abscess#Classification), [respiratory infections](https://en.wikipedia.org/wiki/Respiratory_disease#Respiratory_tract_infections) such as [sinusitis](https://en.wikipedia.org/wiki/Sinusitis), and [food poisoning](https://en.wikipedia.org/wiki/Food_poisoning). S. aureus is one of the leading pathogens for deaths associated with antimicrobial resistance and the emergence of [antibiotic-resistant](https://en.wikipedia.org/wiki/Antibiotic-resistant) strains, such as [methicillin-resistant S. aureus](https://en.wikipedia.org/wiki/Methicillin-resistant_Staphylococcus_aureus) (MRSA), is a worldwide problem in [clinical medicine,](https://en.wikipedia.org/wiki/Medicine#Clinical_practice) can cause a range of illnesses, from minor skin infections, such as [pimples](https://en.wikipedia.org/wiki/Pimple), [impetigo](https://en.wikipedia.org/wiki/Impetigo), [boils](https://en.wikipedia.org/wiki/Boil), [cellulitis](https://en.wikipedia.org/wiki/Cellulitis), [folliculitis](https://en.wikipedia.org/wiki/Folliculitis), [carbuncles](https://en.wikipedia.org/wiki/Carbuncle), [scalded skin syndrome](https://en.wikipedia.org/wiki/Scalded_skin_syndrome), and [abscesses](https://en.wikipedia.org/wiki/Abscess), to life-threatening diseases such as [pneumonia](https://en.wikipedia.org/wiki/Pneumonia), [meningitis](https://en.wikipedia.org/wiki/Meningitis), [osteomyelitis](https://en.wikipedia.org/wiki/Osteomyelitis), [endocarditis](https://en.wikipedia.org/wiki/Endocarditis), [toxic shock syndrome](https://en.wikipedia.org/wiki/Toxic_shock_syndrome), [bacteremia](https://en.wikipedia.org/wiki/Bacteremia), and [sepsis](https://en.wikipedia.org/wiki/Sepsis). It is still one of the five most common causes of [hospital-acquired infections](https://en.wikipedia.org/wiki/Hospital-acquired_infection) and is often the cause of [wound infections](https://en.wikipedia.org/wiki/Complication_(medicine)) following [surgery](https://en.wikipedia.org/wiki/Surgery). mortality rate of 40% has been reported for patients with systemic candidiasis due to C. albicans.
- **Streptococcus species** - responsible for many cases of [pink eye](https://en.wikipedia.org/wiki/Conjunctivitis), meningitis, [bacterial pneumonia](https://en.wikipedia.org/wiki/Bacterial_pneumonia), [endocarditis](https://en.wikipedia.org/wiki/Endocarditis), [erysipelas](https://en.wikipedia.org/wiki/Erysipelas), and [necrotizing fasciitis](https://en.wikipedia.org/wiki/Necrotizing_fasciitis) (the 'flesh-eating' bacterial infections). However, many streptococcal species are not pathogenic, and form part of the [commensal](https://en.wikipedia.org/wiki/Commensalism) human [microbiota](https://en.wikipedia.org/wiki/Microbiota_(microbiology)) of the mouth, skin, intestine, and upper respiratory tract.
- **Serratia marcescens -** Gram-negative bacteria in the family [Yersiniaceae](https://en.wikipedia.org/wiki/Yersiniaceae). It is a [facultative anaerobe](https://en.wikipedia.org/wiki/Facultative_anaerobe) and an opportunistic pathogen in humans, is commonly involved in [hospital-acquired infections,](https://en.wikipedia.org/wiki/Hospital-acquired_infection) particularly [catheter-associated](https://en.wikipedia.org/wiki/Central_venous_catheter#Infection) [bacteremia](https://en.wikipedia.org/wiki/Bacteremia), [urinary tract infections](https://en.wikipedia.org/wiki/Urinary_tract_infection), and [wound infections](https://en.wikipedia.org/wiki/Wound_infection), and is responsible for 1.4% of hospital-acquired infections in the United States. It is commonly found in the [respiratory](https://en.wikipedia.org/wiki/Respiratory_tract) and [urinary tracts](https://en.wikipedia.org/wiki/Urinary_tract) of hospitalized adults and in the [gastrointestinal systems](https://en.wikipedia.org/wiki/Gastrointestinal_system) of children.
