# Supplementary material for: The impact of preoperative oropharyngeal microflora, decontamination, and postoperative nosocomial and opportunistic infections on the occurrence of respiratory complications in patients undergoing esophagectomy for esophageal cancer after chemoradiotherapy. A single-center cohort
Source: Langenbecks Arch Surg. 2026 Jan 17;411(1):70. doi: 10.1007/s00423-026-03966-y (PMC12847148; doi:10.1007/s00423-026-03966-y)
Supplement: Supplementary file 2 — (DOCX 20.8 KB) [file 423_2026_3966_MOESM2_ESM.docx]

| Preoperative cultivation | | Eradication | | | | p-value |
| --- | --- | --- | --- | --- | --- | --- |
|  |  | Yes | | No | |  |
|  |  | No.: | % | No.: | % |  |
| Bacteroidaceae | 0 | 99 | 100.0% | 115 | 99.1% | 1 |
|  | 1 | 0 | 0.0% | 1 | 0.9% |  |
| Staphylococcaceae | 0 | 99 | 100.0% | 115 | 99.1% | 1 |
|  | 1 | 98 | 99.0% | 114 | 97.4% |  |
| Corynebacteriaceae | 0 | 0 | 0.0% | 1 | 0.9% | 1 |
|  | 1 | 1 | 1.0% | 2 | 1.7% |  |
| Pasteurellaceae | 0 | 95 | 96.0% | 113 | 97.4% | 0.706 |
|  | 1 | 4 | 4.0% | 3 | 2.6% |  |
| Enterobacteriaceae | 0 | 94 | 94.9% | 106 | 91.4% | 0.422 |
|  | 1 | 5 | 5.1% | 10 | 8.6% |  |
| Saccharomycetaceae | 0 | 97 | 98.0% | 114 | 98.3% | 1 |
|  | 1 | 2 | 2.0% | 2 | 1.7% |  |
| Streptokok | 0 | 98 | 99.0% | 112 | 96.6% | 1 |
|  | 1 | 1 | 1.0% | 4 | 3.5% |  |
| Moraxellaceae | 0 | 98 | 99.0% | 116 | 100.0% | 0.460 |
|  | 1 | 1 | 1.0% | 0 | 0.0% |  |
| Neisseriaceae | 0 | 99 | 100.0% | 115 | 99.1% | 1 |
|  | 1 | 0 | 0.0% | 1 | 0.9% |  |
| Pasteurellaceae | 0 | 92 | 92.9% | 109 | 94.0% | 0.759 |
|  | 1 | 7 | 7.1% | 7 | 6.0% |  |
| Streptococcaceae | 0 | 90 | 90.9% | 105 | 90.5% | 0.921 |
|  | 1 | 9 | 9.1% | 11 | 9.5% |  |
| Yersiniaceae | 0 | 99 | 100.0% | 115 | 99.1% | 1 |
|  | 1 | 0 | 0.0% | 1 | 0.9% |  |
| Saccharomycetaceae | 0 | 89 | 89.9% | 103 | 88.8% | 0.794 |
|  | 1 | 10 | 10.1% | 13 | 11.2% |  |
| Enterobacteriaceae family | 0 | 93 | 93.9% | 102 | 87.9% | 0.131 |
|  | 1 | 6 | 6.1% | 14 | 12.1% |  |
| Staphylococcus aureus | 0 | 97 | 98.0% | 116 | 100.0% | 0.211 |
|  | 1 | 2 | 2.0% | 0 | 0.0% |  |
| Moraxellaceae | 0 | 95 | 96.0% | 116 | 100.0% | **0.043** |
|  | 1 | 4 | 4.0% | 0 | 0.0% |  |
| Hafniaceae | 0 | 98 | 99.0% | 116 | 100.0% | 0.460 |
|  | 1 | 1 | 1.0% | 0 | 0.0% |  |
| Pseudomonadaceae family | 0 | 98 | 99.0% | 114 | 98.3% | 1 |
|  | 1 | 1 | 1.0% | 2 | 1.7% |  |

Preoperative oropharyngeal culture yielded 12 strains while sputum culture captured 19 strains Among these, the Moraxellaceae family showed statistical significance in the eradication group (p=0.043) for eradication, whereas E. coli showed statistical significance in the non-eradication group as non eradicated.

| Preoperative cultivation | | Without eradication | | | | p-value |
| --- | --- | --- | --- | --- | --- | --- |
|  |  | No | | Yes | |  |
|  |  | Count | Row N % | Count | Row N % |  |
| E. colli | 0 | 99 | 100.0% | 109 | 94.0% | **0.016** |
|  | 1 | 0 | 0.0% | 7 | 6.0% |  |
